# Biocatalytic synthesis of the Green Note *trans*-2-hexenal in a continuous-flow microreactor

**DOI:** 10.3762/bjoc.14.58

**Published:** 2018-03-26

**Authors:** Morten M C H van Schie, Tiago Pedroso de Almeida, Gabriele Laudadio, Florian Tieves, Elena Fernández-Fueyo, Timothy Noël, Isabel W C E Arends, Frank Hollmann

**Affiliations:** 1 Department of Biotechnology, Delft University of Technology, Van der Maasweg 9, 2629 HZ Delft, The Netherlands; 2 Department of Chemical Engineering and Chemistry, Micro Flow Chemistry & Process Technology, Eindhoven University of Technology, Den Dolech 2, 5612 AZ Eindhoven, The Netherlands; 3 Centro de Investigaciones Biológicas, CSIC, Madrid, Spain

**Keywords:** alcohol oxidase, alcohol oxidation, aldehyde, flow chemistry

## Abstract

The biocatalytic preparation of *trans*-hex-2-enal from *trans*-hex-2-enol using a novel aryl alcohol oxidase from *Pleurotus eryngii* (*Pe*AAOx) is reported. As O_2_-dependent enzyme *Pe*AAOx-dependent reactions are generally plagued by the poor solubility of O_2_ in aqueous media and mass transfer limitations resulting in poor reaction rates. These limitations were efficiently overcome by conducting the reaction in a flow-reactor setup reaching unpreceded catalytic activities for the enzyme in terms of turnover frequency (up to 38 s^−1^) and turnover numbers (more than 300000) pointing towards preparative usefulness of the proposed reaction scheme.

## Introduction

*trans*-2-Hexenal is well-known as a major component of the Green Notes of fruits and vegetables such as apples, strawberries, cherries and more. It is widely used in the flavour and fragrance industry as fresh flavour ingredient in foods and beverages.

**Scheme 1:**
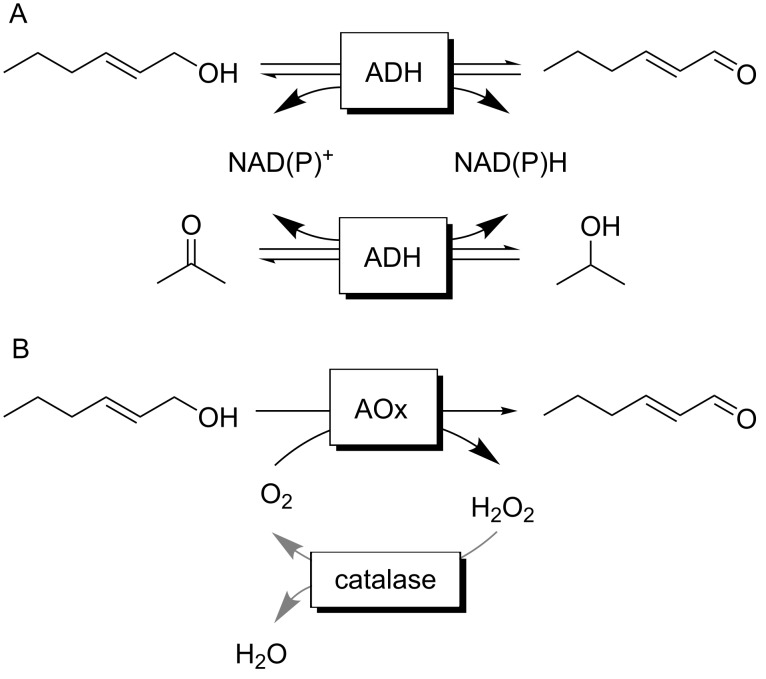
Enzymatic reaction schemes for the selective oxidation of *trans*-hex-2-enol. A: Alcohol dehydrogenase (ADH)-catalysed oxidation producing stoichiometric amounts of NAD(P)H, which needs to be recycled in situ; the overall reaction is reversible requiring surpluses of the cosubstrate (e.g., acetone) to shift the overall equilibrium to the side of *trans*-hex-2-enal. B: Envisioned aerobic oxidation using alcohol oxidases (AOx). H_2_O_2_ is formed as byproduct and dismutated by catalase into H_2_O and O_2_.

One attractive access to *trans*-2-hex-2-enal is the oxidation of the corresponding allylic alcohol to the aldehyde. Though at first sight an oxidation of primary alcohols to the corresponding aldehydes does not appear to be a major challenge, the methods of the state-of-the-art are mostly plagued by undesired side reactions [1]. Also some of the stoichiometric oxidants used are questionable from an environmental and/or toxicological point of view and therefore are not compatible with consumer products such as Green Notes. Therefore, we turned our attention to biocatalytic oxidation methods. For clean conversion of primary alcohols to aldehydes principally two biocatalytic approaches are available (Scheme 1) [2–5]. Alcohol dehydrogenases catalyse the reversible oxidation of alcohols in a Meerwein–Ponndorf–Verley-type of reaction (Scheme 1A). The poor thermodynamic driving force of this reaction, however, necessitates significant molar surpluses of the stoichiometric oxidant (such as acetone). This not only negatively influences the environmental impact of the reaction [6] but also complicates downstream processing. Furthermore, the nicotinamide cofactor (even if used in catalytic amounts only) causes additional costs.

Therefore, we concentrated on alcohol oxidase-catalysed reaction schemes (Scheme 1B. Oxidases utilise O_2_ as terminal electron acceptor for the oxidation reaction yielding H_2_O_2_ as sole byproduct. The latter can be disproportionated easily by using catalase (Scheme 1B). Furthermore, O_2_ reduction adds sufficient thermodynamic driving force to the reaction to make it essentially irreversible.

The benefits of using O_2_, however, also come with the disadvantage of its very poor solubility in aqueous media (ca. 0.25 mM at room temperature). Hence, in the course of an oxidation reaction dissolved O_2_ is consumed rapidly and diffusion of O_2_ into the reaction medium can easily become overall rate-limiting. The O_2_ diffusion rate into the reaction medium directly correlates with the interfacial area between aqueous medium and the gas phase. Large interfacial surface areas can be achieved via heterogeneous intake, by bubbling, stirring, etc. Soluble enzymes, however, are often rather unstable under these conditions, possibly owing to the mechanical stress leading to irreversible inactivation of the biocatalyst [7–8]. Methods of bubble-free aeration have been described in the literature to alleviate the inactivation issue described above [9–12].

The continuous-flow microreactor technology has emerged as a safe and scalable way to approach oxidation reactions [13–14]. Due to its small dimensions, hazardous reactions can be easily controlled, owing to the large surface-to-volume ratio which can minimise hot-spot formation and allows for control over mixing and heating phenomena [15–16]. Furthermore, a well-defined gas–liquid regime can be easily maintained [17–18]. High mass-transfer coefficients are generally the consequence of small vortices induced by the segmented flow regime. This flow pattern guarantees an enhanced contact between the two phases and provides a uniform gas concentration in the liquid segment.

Therefore, it is not very astonishing that also the biocatalysis community is showing interest in flow chemistry. Several biocatalytic processes have been reported in flow reactors [19], mostly advocating easier process intensification in combination with enzyme immobilization [20–23]. Also the higher oxygen-transfer rates in flow reactions compared to batch reactions have been emphasised by several groups. Here, reactor designs ranging from simple flow reactors, tube-in-tube reactors [24], agitated tube reactors [25–26] and continuous agitated cell reactors [27] have been reported.

Encouraged by these contributions, we asked ourselves whether a slug-flow approach may combine mechanically less demanding conditions with high O_2_-transfer rates thereby enabling efficient and robust oxidase-catalysed oxidation reactions.

## Results and Discussion

### Selection and characterisation of the biocatalyst

**Figure 1:**
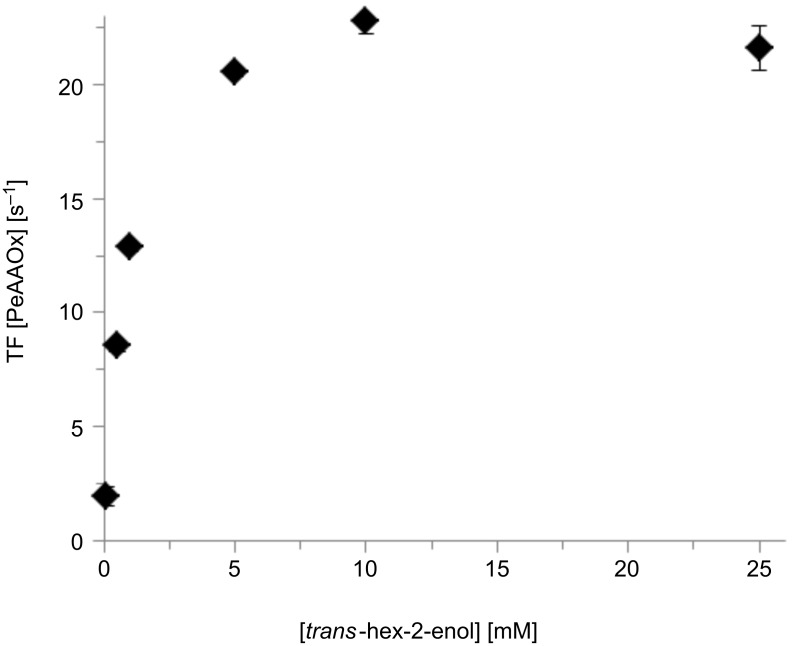
Michaelis–Menten kinetics of the *Pe*AAOx-catalysed oxidation of *trans*-hex-2-enol. Conditions: 50 mM KP_i_ buffer (pH 7, 30 °C), [*trans*-hex-2-enol]_0_ = 3 mM, [*Pe*AAOx] = 0.044 µM, [horseradish peroxidase] = 500 U mL^−1^, [ABTS] = 2 mM.

As biocatalyst for this study we focussed on the recombinant aryl alcohol oxidase from *Pleurotus eryngii* (*Pe*AAOx) [28–31]. Especially the availability as recombinant enzyme (enabling future at-scale production and protein engineering) and its promising activity on allylic alcohols make *Pe*AAOx a promising starting point. Commercially available alcohol oxidases from *Pichia pastoris* and *Candida boidinii* showed no significant activity for the substrate under the same conditions. As *trans*-2-hex-enol had not been reported as substrate for *Pe*AAOx we evaluated its catalytic properties, particularly the substrate concentration-dependency of the enzymatic oxidation. Initial rate measurements (performed in 1 mL cuvettes) revealed a Michaelis–Menten dependency of the enzyme activity (Figure 1). Apparent *K*_M_ and *k*_cat_ values of approximately 1 mM and 22 s^−1^ were estimated, respectively. These values are in the same order of magnitude as those for benzyl alcohol substrates reported previously [29]. The slightly decreasing enzyme activity at elevated substrate concentrations may be an indication for a slight substrate inhibition. Performing these initial rate measurements in the presence of varying product concentrations showed a pronounced product inhibition (Supporting Information File 1, Figure S2, vide infra).

### Continuous-flow reactor enzymatic oxidation

**Figure 2:**
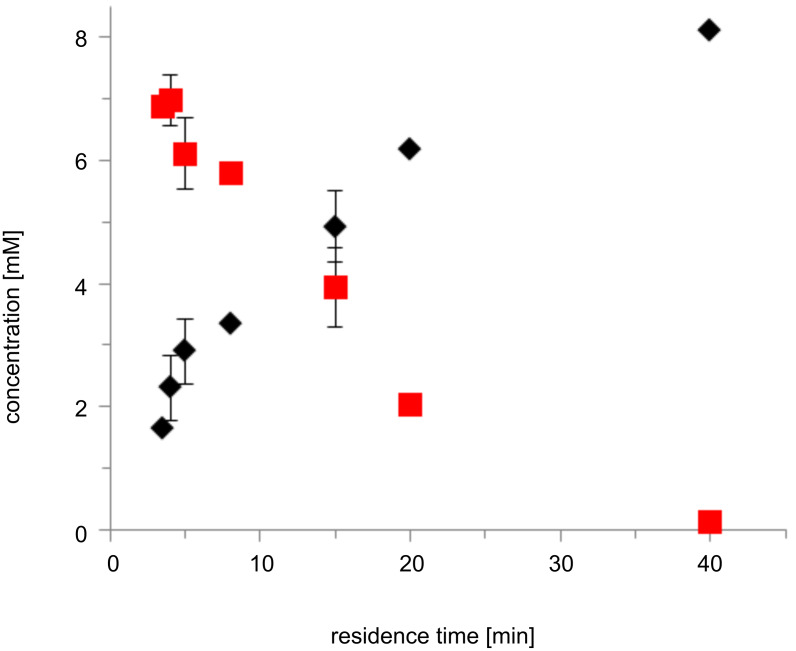
The influence of the residence time on the conversion of *trans*-hex-2-enol (red squares) to *trans*-2-hexenal (black diamonds) in a flow reactor. Conditions: 3 mL flow reactor, 50 mM KP_i_ buffer (pH 7, 30 °C), [*trans*-hex-2-enol]_0_ = 10 mM, [*Pe*AAOx] = 0.25 µM, [catalase] = 600 U mL^−1^.

Next, we performed the *Pe*AAOx-catalysed oxidation of *trans*-hex-2-enol in a slug-flow reactor setup (Supporting Information File 1, Figure S1 and Figures S9–S11). In a first set of experiments we systematically varied the residence time of the reaction mixture in the flow reactor (and thereby the reaction time, Figure 2).

A full conversion of the starting material into the desired *trans*-hex-2-enal was observed at residence (reaction) times of approximately 40 min corresponding to a turnover number (TN) for the biocatalysts of 32400 and an average turnover frequency (TF) of 13.5 s^−1^. Even more interestingly, at higher flow rates apparent TF of up to 38 s^−1^ (RT = 5 min) were observed. This value exceeds the previously determined *k*_cat_(*Pe*AAOx) (Figure 1) significantly. We attribute this observation to an increased oxygen-transfer rate at high flow rates. In the case of the 5 minutes residence time this corresponds to an O_2_-transfer rate of roughly 0.25 mM min^−1^. Similarly high values could be obtained previously only under mechanically demanding reaction conditions or using surfactant-stabilised emulsions [7]. Varying the ratio of gas to liquid had no significant effect on the overall rate of the reaction (Table 1).

**Table 1:** Effect of variation of the gas-to-liquid ratio on the rate of the *Pe*AAOx-catalysed aerobic oxidation of *trans*-hex-2-enol.

ratio (liquid:gas)	liquid flow [mL min^−1^]	gas flow [mL min^−1^]	residence time [min]	[product] [mM]

1:1	0.20	0.20	15	5.48 (± 0.01)
1:3	0.10	0.30	15	5.18 (± 0.32)
1:5	0.067	0.333	16	4.99 (± 0.49)

Conditions: 3 mL flow reactor, 50 mM KP_i_ buffer (pH 7, 30 °C), [*trans*-2-hexen-1-ol]_0_ = 10 mM, [*Pe*AAOx] = 0.25 µM, [catalase] = 600 U mL^−1^.

Within the experimental error, the conversion in all experiments was identical indicating that even at a comparably low volumetric ratio of 1:1 the O_2_ availability was already sufficient not to be overall rate-limiting.

It is worth mentioning here that under batch reaction conditions, similar progression curves were only attainable under mechanically very demanding conditions (i.e., very vigorous stirring and bubbling of O_2_ directly into the reaction mixture, Supporting Information File 1, Figure S3). These conditions also caused a significant evaporation of the substrate at higher substrate concentration (Supporting Information File 1, Figure S4), which was much less the case in the flow-reaction setup.

**Figure 3:**
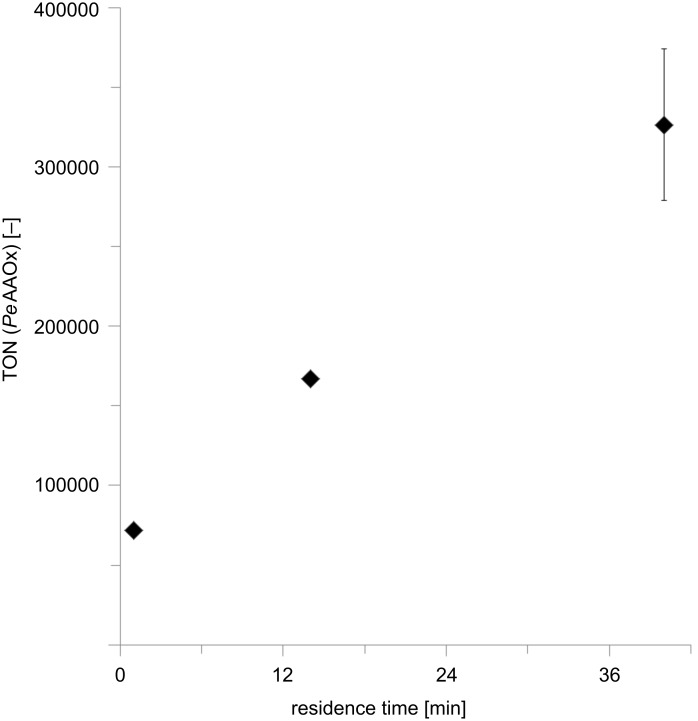
Increasing the *Pe*AAOx turnover numbers (TN) by increasing the residence time. Conditions: 6 mL flow reactor, 50 mM KP_i_ buffer (pH 7, 30 °C), [*trans*-hex-2-enol]_0_ = 40 mM, [*Pe*AAOx] = 0.02 µM, [catalase] = 600 U mL^−1^. The TN value was calculated based on the GC yield of every run. The TN was obtained by dividing the product concentration (as determined chromatographically) by the biocatalyst concentration.

From an economical point-of-view the catalyst performance in terms of turnover number (TN) is of utmost importance as it directly correlates with the cost-contribution of the catalyst to the production costs [32–34]. Therefore we evaluated the TN attainable for *Pe*AAOx in the flow setup (Figure 3). For this lower *Pe*AAOx concentrations as well as significantly increased residence times were applied. The increased residence times were achieved by decreasing the flow rates and using a longer flow reactor (6 mL volume instead of 3 mL).

Pleasingly, already in these first experiments a TN for the enzyme of more than 300000 was observed at long residence times. This also underlines the robustness of the enzyme under the flow conditions. Compared to Figure 2 somewhat lower TFs for *Pe*AAOx were observed here, which again can be attributed to a lower O_2_-transfer rate at lower flow rates. The quasi-linear relationship shown in Figure 3 also suggests that even higher TN may be attainable – however at the expense of longer reaction times. Therefore, further investigations will focus on identifying conditions satisfying the demand for high TNs and short reaction times. Encouraged by these results, we also tried a semi-preparative scale reaction using 5 g L^−1^ (50 mM) substrate loading in a total of 50 mL with 0.75 μM *Pe*AAOx. As a result, 90% conversion was achieved after 18 h of total reaction time (roughly 80 minutes of residence time in the 6 mL reactor). The product was purified chromatographically resulting in 200 mg of pure *trans*-hex-2-enal (as determined by NMR) in 81% isolated yield thereby demonstrating the preparative potential of the proposed reaction setup.

## Conclusion

Alcohol oxidase-catalysed oxidation of alcohols to aldehydes bears a significant potential for preparative biocatalysis. The reaction is independent from expensive and instable nicotinamide cofactors (and the corresponding cosubstrates/coproducts as well as possible regeneration enzymes) and produces only water as byproduct. These advantages, however, are counteracted by the generally low reaction rates caused by the poor O_2_ availability. Flow chemistry is a promising technique to provide the aqueous reaction mixture with O_2_ needed for the oxidation. It enables high O_2_ transfer rates while avoiding enzyme robustness issues frequently observed with ‘traditional’ aeration methods.

Future developments in our laboratories will concentrate on the characterisation, extension and preparative demonstration of this powerful combination of oxidase catalysis and flow chemistry.

## Experimental

### General

Turnover numbers (TN) and turnover frequencies (TF) reported in this manuscript were calculated based on Equation 1 and Equation 2.

[1]
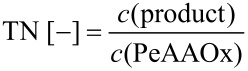


[2]
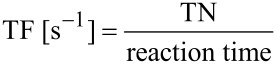


### Production of *Pe*AAOx

#### *E. coli* cultivation

For the production, activation and purification of *Pe*AAOx, a slightly modified literature protocol was used [28]. Pre-cultures of LB media containing 100 μg mL^−1^ of ampicillin were inoculated with *E. coli* W3110 containing pFLAG1-AAO and incubated overnight at 37 °C and 180 rpm. Overexpression was carried out in 5 L flasks with 1 L of TB medium supplemented with 100 μg mL^−1^ of ampicillin. The medium was inoculated with the pre-culture to an OD of 0.05 and grown at 37 °C and 180 rpm. At an OD_600_ of 0.8, 1 mM isopropyl β-D-thiogalactopyranoside (IPTG) was added and the cultures were incubated for additional 4 h at 37 °C and 180 rpm. The bacterial pellets, obtained after harvesting the cells, were re-suspended in a total volume of 40 mL 50 mM Tris/HCl buffer, pH 8.0, containing 10 mM EDTA and 5 mM dithiothreitol (DTT).

#### Refolding

The re-suspended cells were disrupted by incubation with 2 mg mL^−1^ lysozyme for 1 h at 4 °C. Afterwards, 0.1 mg mL^−1^ DNase, 1 mM MgCl_2_ and 0.1 mM PMSF were added followed by sonication. The insoluble fraction was collected by centrifugation (30 min at 15,000 rpm and 4 °C), re-suspended and washed three times with 20 mL 20 mM Tris/HCl buffer, pH 8.0, containing 10 mM EDTA and 5 mM DTT using a potter homogenizing device. The pellets obtained after centrifugation (15 min at 15,000 rpm and 4 °C) were solubilized in a total volume of 30 mL 20 mM Tris/HCl buffer, pH 8.0, containing 2 mM EDTA, 50 mM DTT and 8 M urea. After incubation on ice for 30 min, the solution was cleared by centrifugation (15 min at 15,000 rpm and 4 °C). The obtained supernatant was used as stock solution for the in vitro refolding.

The *Pe*AAOx was solubilized using 150 µg mL^−1^ protein in 20 mM Tris/HCl buffer, pH 9.0, containing 2.5 mM GSSG, 1 mM DTT, 0.02 mM FAD, 34% glycerol and 0.6 M urea at 4 °C for 80 h. After the incubation for *Pe*AAOx activation/refolding, the refolding mixture was concentrated to 100 mL and the buffer exchanged against 10 mM sodium phosphate buffer, pH 5.5 by diafiltration (DV 20) and subsequently concentrated using an Amicon Ultra 15 mL centrifugal filter (MWCO 10 kDa). After centrifugation (overnight at 15,000 rpm and 4 °C), the soluble fraction was further purified using anion-exchange chromatography.

#### Purification

The concentrated *Pe*AAOx solution was purified using a 58 mL Q Sepharose column (GE Healthcare). *Pe*AAOx was eluted with a linear NaCl gradient (0–0.6 M over 6 CV) using 10 mM sodium phosphate buffer, pH 5.5. Fractions containing *Pe*AAOx were pooled, concentrated and desalted using HiTrap desalting columns (GE Healthcare) and 10 mM sodium phosphate buffer, pH 5.5. The *Pe*AAOx concentration was calculated based on the absorbance using the molar extinction coefficient of ε_463_ 11,050 M^−1^ cm^−1^.

### Activity assay

The activity of *Pe*AAOx was determined by UV–vis spectroscopy, using an Agilent Cary 60 UV–vis spectrophotometer, following the oxidation of ABTS (ε_405_ = 36,800 M^−1^ cm^−1^) by horseradish peroxidase (POD) at the expense of hydrogen peroxide. In general, 0.044 µM *Pe*AAOx was used to convert 3 mM of *trans*-2-hex-2-enol. The hydrogen peroxide formed in this reaction was subsequently used to convert 2 mM of ABTS to ABTS^·+^ by an excess of POD (500 U mL^−1^). The reactions were performed at 30 °C in oxygen-saturated 50 mM KP_i_ buffer at pH 7.0.

### Flow reactor experiments

PFA microreactor coils (750 μm ID) with a volume of 3 and 6 mL were constructed. The reaction mixture was introduced via a syringe pump (Fusion 200, Chemyx), while the pure oxygen flow was controlled by a mass flow controller (EL-FLOW, Bronkhorst), resulting in a segmented flow (Supporting Information File 1, Figure S8). Residence times were taken as the time between the solution entering and exiting the coil and were varied by altering the flow, keeping the ratio of oxygen to liquid at three to one. Samples were collected on ice and as soon as enough volume was collected, extracted with ethyl acetate and analysed by GC (vide infra).

### GC analysis

The collected reaction mixtures were extracted into an equal volume of ethyl acetate, dried with magnesium sulphate and analysed on a CP-wax 52 CB GC column (50 m × 0.53 m × 2 µm) (GC method: 60 °C for 3 min; 30 °C/min to 105 °C; 105 °C for 7 min; 30 °C/min to 250 °C; 250 °C for 1 minute). Dodecane (5 mM) was added as standard.

### Work-up semi-preparative scale

The reaction mixture was directly collected in deuterated chloroform at the end of the flow reactor followed by recording the NMR spectrum in order to evaluate the conversion (see Supporting Information File 1). The organic mixture was diluted and introduced into a separation funnel and washed with brine. The aqueous phase was backwashed once with DCM. The collected organic phase was dried over MgSO_4_, filtered and concentrated under reduced pressure. Purification of the isolated mixture was performed by flash chromatography on silica (pure DCM). The final product was obtained as colourless oil (200 mg).

#### (*E*)-Hex-2-enal


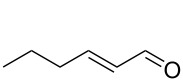


TLC (DCM) *R*_f_ 0.9; ^1^H NMR (399 MHz, CDCl_3_) δ 9.44 (d, *J* = 7.7 Hz, 1H), 6.78 (dt, *J* = 15.6, 6.8 Hz, 1H), 6.05 (ddq, *J* = 15.5, 7.8, 1.3 Hz, 1H), 2.33–2.18 (m, 2H), 1.48 (h, *J* = 7.4 Hz, 2H), 0.90 (t, *J* = 7.4 Hz, 3H); ^13^C NMR (101 MHz, CDCl_3_) δ 194.3, 158.9, 133.3, 34.8, 21.3, 13.8.

## Supporting Information

File 1 General information and supporting figures.
