# Supplementary material for: Biocatalytic synthesis of the Green Note trans-2-hexenal in a continuous-flow microreactor
Source: Beilstein J Org Chem. 2018 Mar 26;14:697–703. doi: 10.3762/bjoc.14.58 (PMC5905246; doi:10.3762/bjoc.14.58)
Supplement: File 1 — General information and supporting figures. [file Beilstein_J_Org_Chem-14-697-s001.pdf]

Supporting Information

for

Biocatalytic synthesis of the Green Note *trans*-2-hexenal in a continuous-flow microreactor

Morten M.C.H. van Schie[1], Tiago Pedroso de Almeida[1], Gabriele Laudadio[2], Florian Tieves[1], Elena Fernández-Fueyo[3], Timothy Noël[2]*, Isabel W.C.E. Arends[1], and Frank Hollmann*[1]

Address: [1]Department of Biotechnology, Delft University of Technology, Van der Maasweg 9, 2629 HZ Delft, The Netherlands, [2]Department of Chemical Engineering and Chemistry, Micro Flow Chemistry & Process Technology, Eindhoven University of Technology, Den Dolech 2, 5612 AZ Eindhoven, The Netherlands and [3]Centro de Investigaciones Biológicas, CSIC, Madrid, Spain

Email: Frank Hollmann - f.hollmann@tudelft.nl; Timothy Noël - t.noel@tue.nl

*Corresponding author

General information and supporting figures

1. General information

All reagents and solvents were used as received without further purification, unless stated otherwise. Technical solvents were purchased from VWR International and Biosolve and used as received. All capillary tubing and microfluidic fittings were purchased from IDEX Health & Science. Used syringes were of BD Discardit II® or NORM-JECT®, purchased from VWR Scientific. Syringe pumps were purchased from Chemix Inc. model Fusion 200 Touch. The mass flow controller (EL-FLOW) was purchased from Bronkhorst. A schematic representation of the setup is shown in Figure S1.

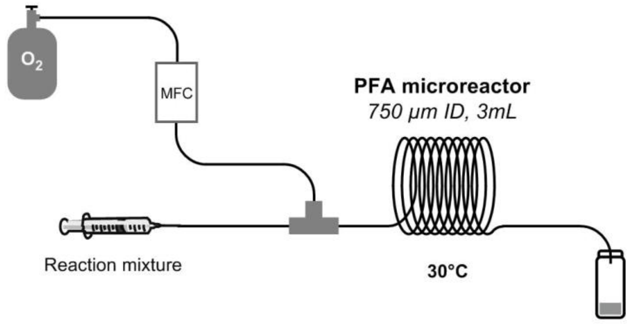

Figure S1: Schematic view of the reaction setup. The oxygen gas flow was controlled by a mass flow controller (MFC). The reaction mixture flow was controlled by a syringe pump. The sample was collected on ice or directly in ethyl acetate at the end of the reactor.

The product isolation was performed automatically by a Biotage® Isolera Four, with Biotage® SNAP KP-Sil 10 or 25 g flash chromatography cartridges. TLC analysis was performed using silica on aluminum foils TLC plates (F254, Supelco Sigma-

Aldrich™) with visualization under ultraviolet light (254 nm and 365 nm) or appropriate TLC staining. ^{1}H (400 MHz) and ^{13}C (100 MHz) NMR spectra were recorded at ambient temperature using a Bruker-Avance 400 or Mercury 400. ^{1}H NMR spectra are reported in parts per million (ppm) downfield relative to $CDCl_3$ (7.26 ppm) and ^{13}C NMR spectra are reported in ppm relative to $CDCl_3$ (77.2 ppm) unless stated otherwise. NMR signals multiplicities are reported using the following abbreviations: s = singlet, d = doublet, t = triplet, q = quartet, p = pentet, h = hextet, hept = heptet, m = multiplet, dd = double doublet, td = triple doublet. NMR data was processed using the MestReNova 9.0.1 software package. Known products were characterized by comparison with the corresponding ^{1}H NMR and ^{13}C NMR reported in the literature. GC analyses were performed on a Shimadzu GC-2014 with an auto sampler unit (AOC-20i). The names of all products were generated using the PerkinElmer ChemBioDraw Ultra v.12.0.2 software package.

2. Supporting figures

2.1 Initial rate experiments evaluating PeAAOx inhibition by *trans*-hex-2-enal

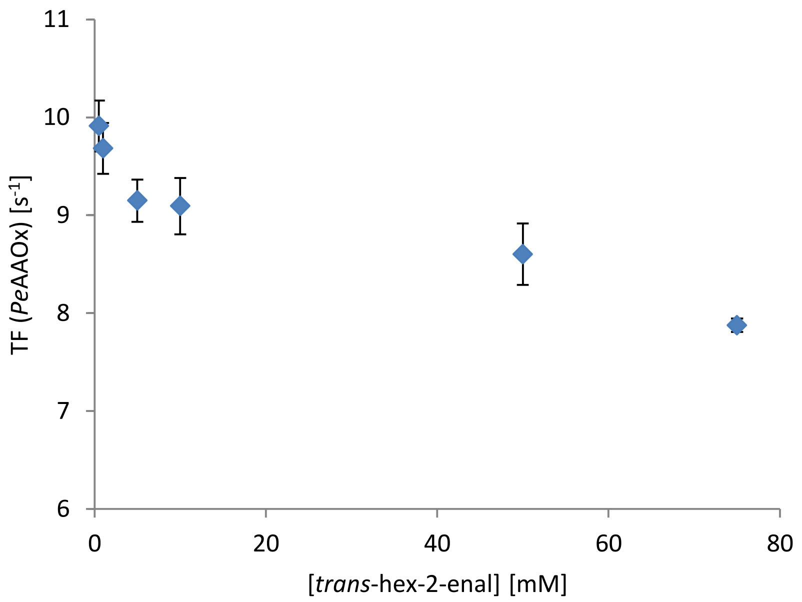

Figure S2: The residual activity of *Pe*AAOx in the presence of the product *trans*-hex-2-enal. Conditions: 50 mM KP_i buffer (pH 7, 20 °C), [*trans*-hex-2-enol]$_0$ = 3 mM, [*Pe*AAOx] = 0.044 µM, [horseradish peroxidase] = 500 U ml^{-1}, [ABTS] = 2 mM.

2.2 Comparative transformation in batch mode under vigurous strirring and bubbling

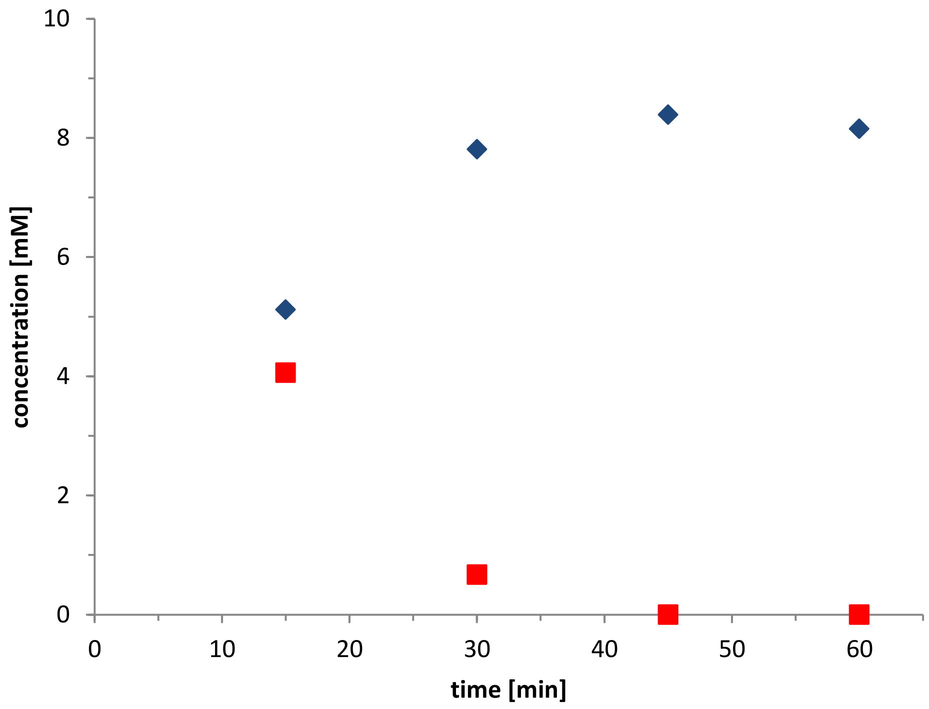

Figure S3: The conversion of *trans*-hex-2-enol (■) to *trans*-2-hex-enal (◆) in a batch reactor while vigorously stirring and bubbling oxygen through the solution. Conditions: 5 ml batch reaction, 50 mM KP_i buffer (pH 7, 30 °C), $[\text{trans-hex-2-enol}]_0$ = 10 mM, [*Pe*AAOx] = 0.25 µM, [catalase] = 600 U ml^{-1}, stirring at 1000 rpm, pure oxygen supplied by a balloon.

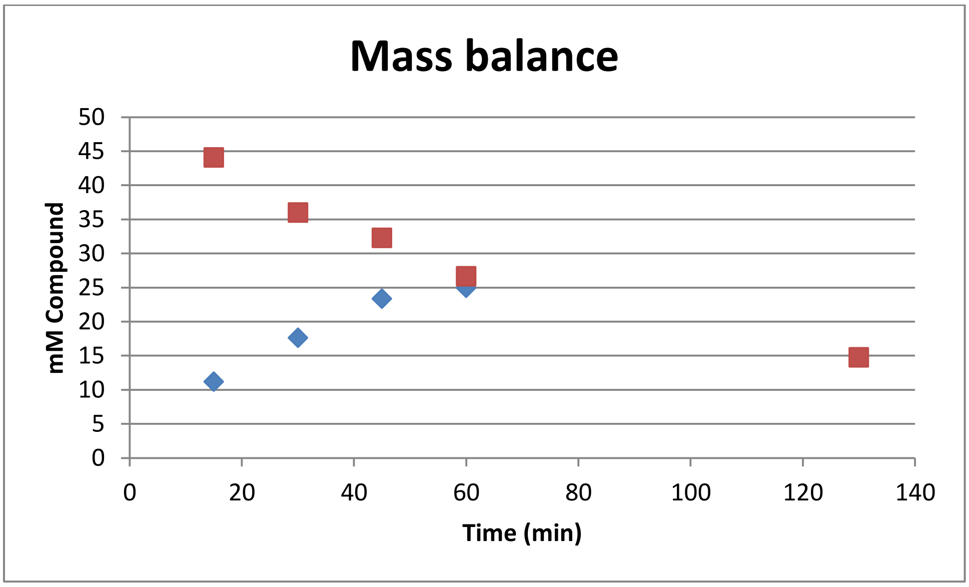

Figure S4: The conversion of 50 mM of *trans*-hex-2-enol to *trans*-2-hex-enal (◆) in a batch reactor, while vigorously stirring and bubbling oxygen through the solution. The mass balance over time is also shown (■).

Conditions: 5 ml batch reaction, 50 mM KP_i buffer (pH 7, 30 °C), $[\text{trans-hex-2-enol}]_0$ = 50 mM, [*Pe*AAOx] = 0.25 µM, [catalase] = 600 U ml^{-1}, stirring at 1000 rpm, pure oxygen supplied by a balloon.

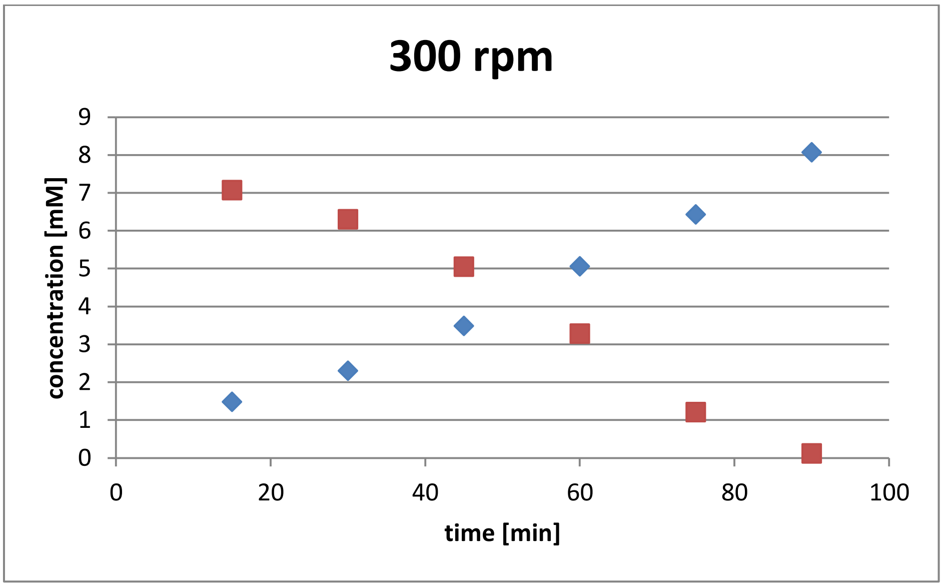

Figure S5: The conversion of *trans*-hex-2-enol (■) to *trans*-2-hex-enal (◆) in a batch reactor, while gently stirring without bubbling oxygen through.

Conditions: 5 ml batch reaction, 50 mM KP_i buffer (pH 7, 30 °C), $[\text{trans-hex-2-enol}]_0$ = 10 mM, [*Pe*AAOx] = 0.25 µM, [catalase] = 600 U ml^{-1}, stirring at 300 rpm, pure oxygen supplied by a balloon.

2.3 NMR spectra of the isolated product

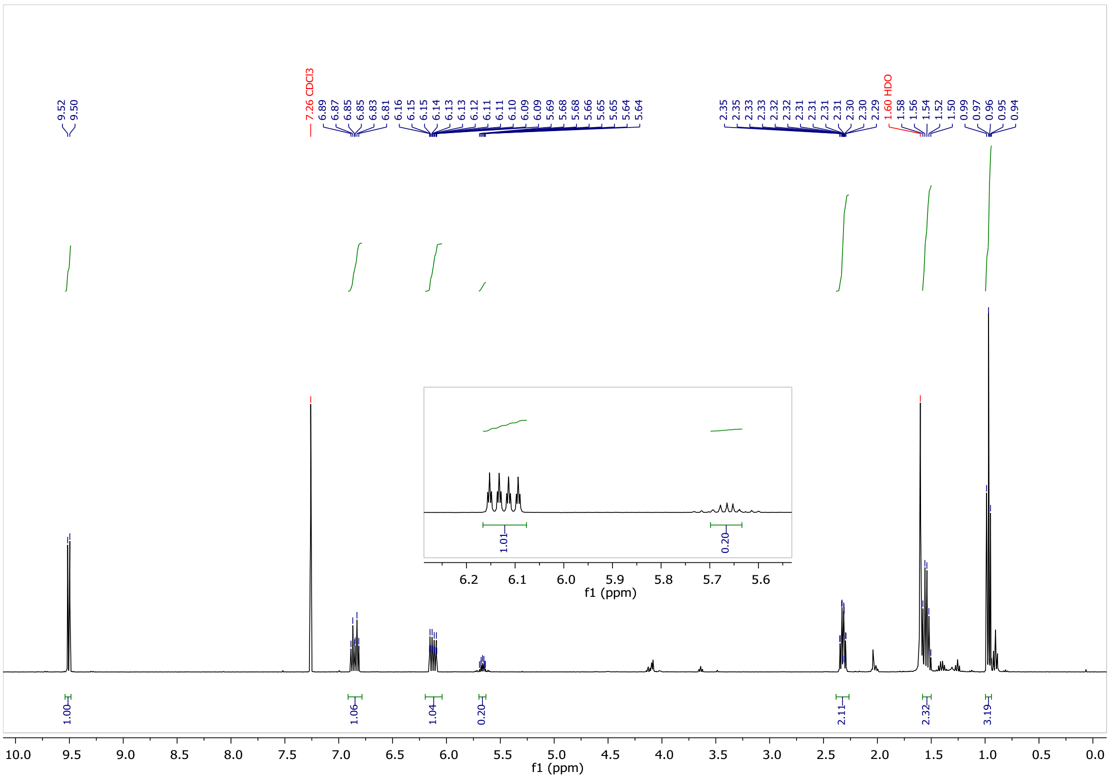

Figure S6: ^{1}H NMR spectrum of the reaction mixture prior to purification.

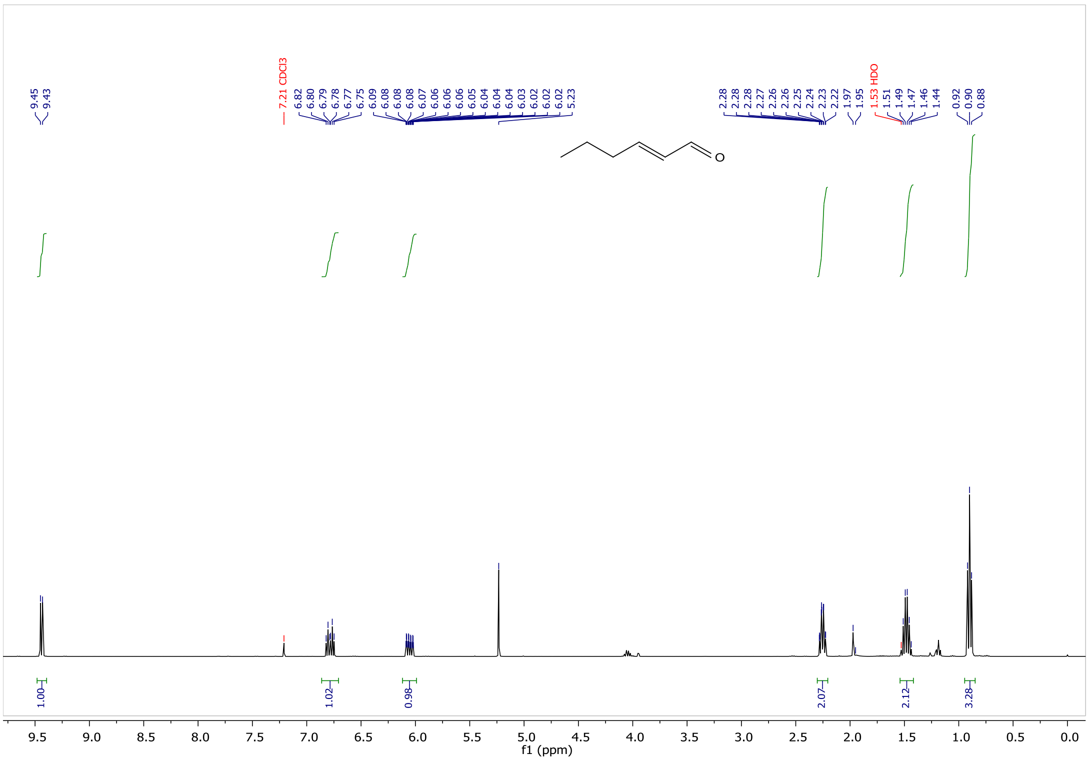

Figure S7: ^{1}H NMR of the product after purification.

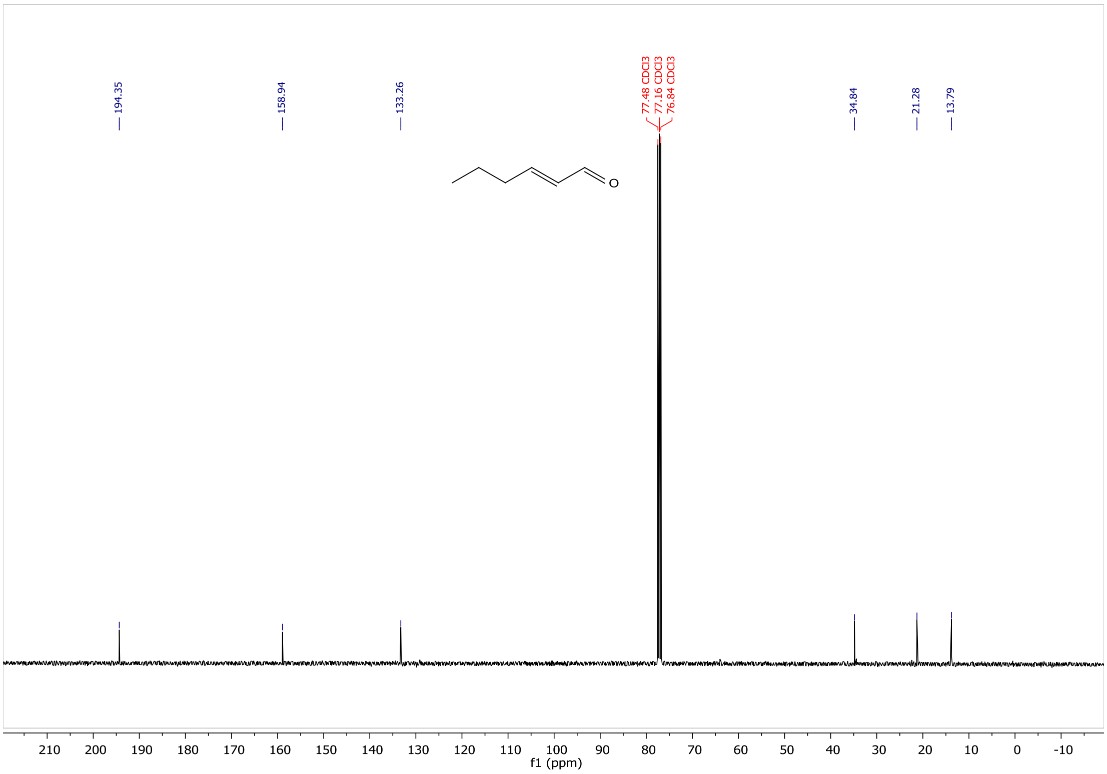

Figure S8: ^{13}C NMR of the product after purification.

2.4 Photographs of the experimental setup

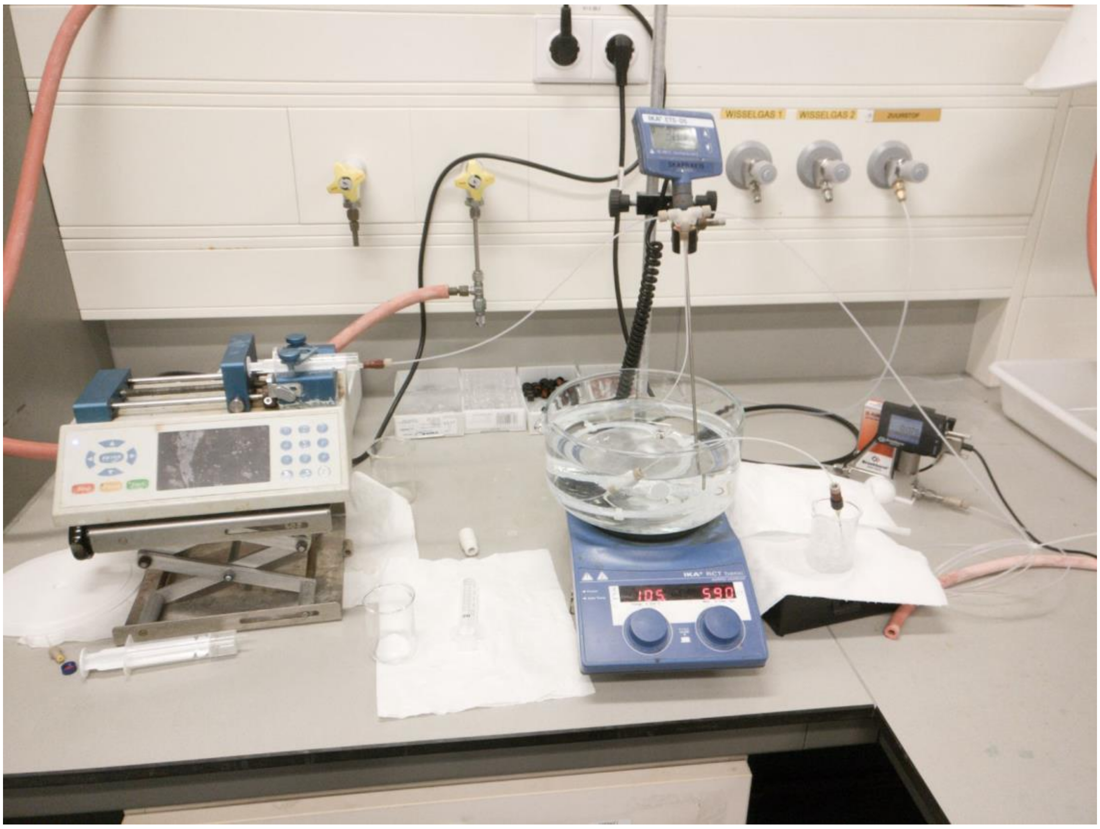

Figure S9: Overview of the flow setup.

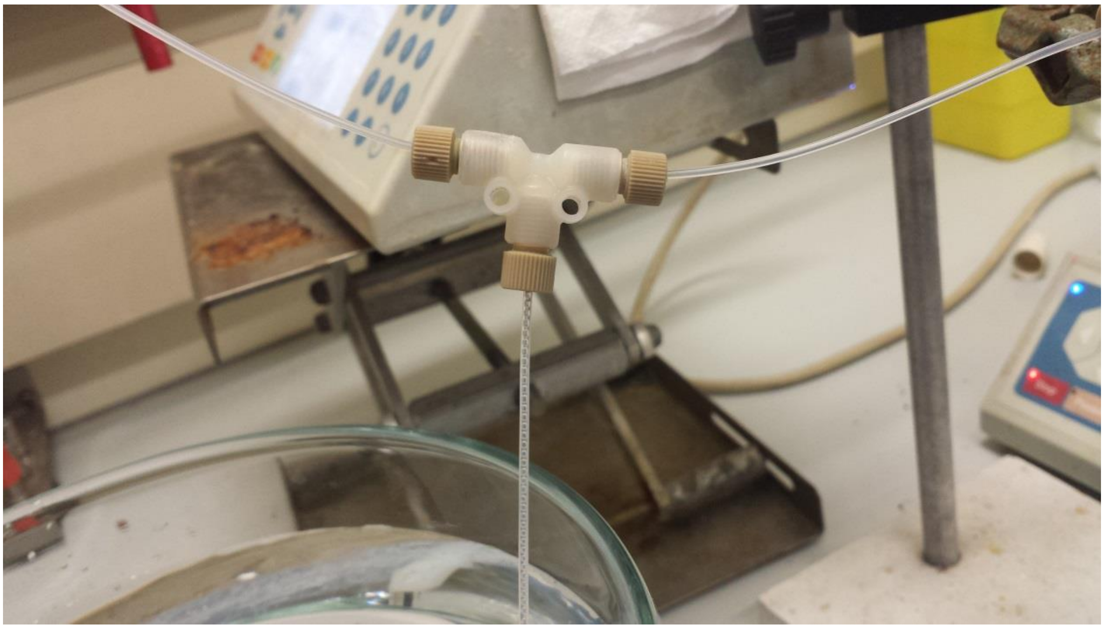

Figure S10: Mixing the two phases.

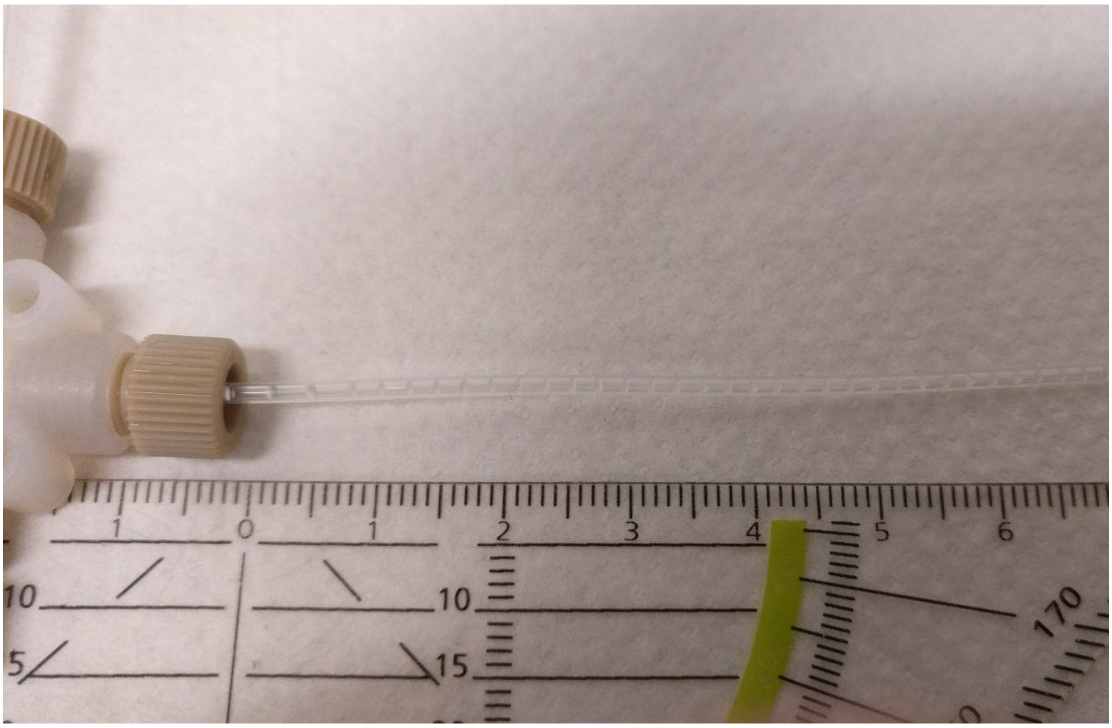

Figure S11: Image of the segmented flow at an oxygen : liquid ratio of three to one at the beginning of the reactor.
